# Biosynthesis and Bioactivity of Prodiginine Analogs in Marine Bacteria, *Pseudoalteromonas*: A Mini Review

**DOI:** 10.3389/fmicb.2019.01715

**Published:** 2019-07-24

**Authors:** Francis E. Sakai-Kawada, Courtney G. Ip, Kehau A. Hagiwara, Jonathan D. Awaya

**Affiliations:** ^1^Department of Molecular Biosciences and Bioengineering, University of Hawai´i at Mānoa, Honolulu, HI, United States; ^2^Department of Biology, University of Hawai´i at Hilo, Hilo, HI, United States; ^3^Institute of Marine and Environmental Technology, University of Maryland, Baltimore, Baltimore, MD, United States; ^4^Chemical Sciences Division, National Institute of Standards and Technology, Hollings Marine Laboratory, Charleston, SC, United States

**Keywords:** *Pseudoalteromonas*, prodiginines, prodigiosin, secondary metabolites, pigments, marine bacteria

## Abstract

The Prodiginine family consists of primarily red-pigmented tripyrrole secondary metabolites that were first characterized in the Gram-negative bacterial species *Serratia marcescens* and demonstrates a wide array of biological activities and applications. Derivatives of prodiginine have since been characterized in the marine γ-proteobacterium, *Pseudoalteromonas*. Although biosynthetic gene clusters involved in prodiginine synthesis display homology among genera, there is an evident structural difference in the resulting metabolites. This review will summarize prodiginine biosynthesis, bioactivity, and gene regulation in *Pseudoalteromonas* in comparison to the previously characterized species of *Serratia*, discuss the ecological contributions of *Pseudoalteromonas* in the marine microbiome and their eukaryotic hosts, and consider the importance of modern functional genomics and classic DNA manipulation to understand the overall prodiginine biosynthesis pathway.

## Introduction

The Prodiginine family consists of primarily red-pigmented secondary metabolites that are characterized by their tripyrrole structure. These metabolites are of interest in natural product research due to their wide array of biomedical and industrial applications including algicidal (Zhang et al., 2016), antibacterial (Okamoto et al., 1998), anticancer (Kawauchi et al., 2008), antimalarial (Kim et al., 1999), antiprotozoal (Azambuja et al., 2004), colorants (Alihosseini et al., 2008), immunosuppressive agents (Kawauchi et al., 2007), and insecticides (Suryawanshi et al., 2015). Prodiginine was first extracted from terrestrial bacterium, *Serratia marcescens*. It consisted of a straight alkyl chain substituent and was named prodigiosin (Rapoport and Holden, 1962). Prodigiosin is ubiquitous throughout various genera within the terrestrial and marine environment, suggesting that the metabolite may be ecologically advantageous to prodiginine-producing bacteria. Due to the diversity among prodiginine-producing bacteria, it is difficult to determine the precise biological and ecological role of the pigment. Based on previous studies, it can be inferred that prodiginine may provide a mode of defense against competing microorganisms or a potential response to environmental stressors (Gallardo et al., 2016).

Since the discovery of prodiginine, analogs have been isolated from marine bacteria, *Pseudoalteromonas*, including cycloprodigiosin and 2-(*p*-hydroxybenzyl) prodigiosin (Rapoport and Holden, 1962; Kawauchi et al., 1997; Fehér et al., 2008). *Pseudoalteromonas* has also been reported to produce tambjamines, a yellow pigment that is structurally like prodiginine, sharing two of the three pyrrole rings (Burke et al., 2007). The presence of pigmented metabolites like prodiginine and tambjamines in species of *Pseudoalteromonas* is particularly interesting because the genus is commonly associated with macroorganisms, such as algae (Dobretsov and Qian, 2002), fish (Pratheepa and Vasconcelos, 2013), sponges (Sakai-Kawada et al., 2016), and tunicates (Holmström et al., 1998). This host-microbe interaction provides the microorganism with nutrients to flourish, while providing the host with valuable resources that may contribute to nutrient cycling or defense (Webster and Taylor, 2012).

Many mechanisms for signaling and regulation of prodigiosin were identified in *Serratia*, however, none have been identified within *Pseudoalteromonas*. It was suggested that prodiginine biosynthesis may prevent primary metabolism overload by utilizing substrates like proline, serine, pyruvate, and NAD(P) (Wasserman et al., 1973; Drummond et al., 1995). PigP was characterized within the quorum sensing regulon as a master regulator of *pig* biosynthesis, controlling prodigiosin production by modulating *pig* regulators (Fineran et al., 2005b) Further studies characterized a metalloregulatory repressor (PigS) that responds to stress-induced presence of heavy metals (Gristwood et al., 2011). Independently, a GntR-homolog (PigT) was also identified (Fineran et al., 2005a). Due to the various means for regulation, it is uncertain if *Pseudoalteromonas* also utilize similar regulatory controls or if it adapted a genus-specific mechanism. Recent studies propose a LuxI/R quorum sensing system associated with pigment production in other *Pseudoalteromonas* species (Pan et al., 2016; Dang et al., 2017).

Although extensive studies were done to determine the bioactivity and chemical structure of these prodiginine analogs, the means of biological synthesis in *Pseudoalteromonas* remains elusive. To date, more than 200 *Pseudoalteromonas* genomes are sequenced and available on genome databases. Of those 200+ genomes, 9 genomes are associated with a prodiginine-producing species and only 3 belong to a tambjamine-producing species.

This review will focus on prodiginine classes that were characterized in *Pseudoalteromonas*, their bioactivity, and their biosynthesis. These ideas may provide further insight into the role prodiginines play in *Pseudoalteromonas*, their hosts, and the marine microbiome.

### Biosynthesis and Bioactivity of Prodiginine Pigments

**FIGURE 1:**
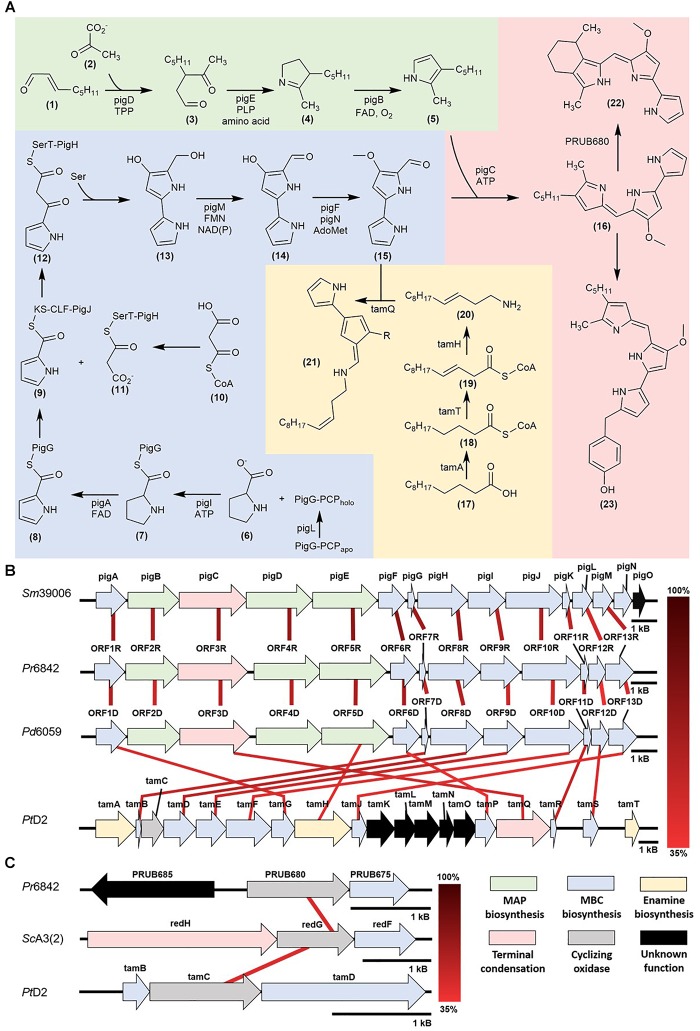
**(A)** Prodiginine biosynthetic pathways: MAP biosynthesis (green), MBC biosynthesis (blue), prodigiosin, cPrG, and HBPG biosynthesis (red), enamine and tambjamine biosynthesis (yellow) **(B)** Mapping of homologous prodiginine biosynthetic gene clusters in *Serratia marcescens* (*Sm*39006), *Pseudoalteromonas* *rubra* (*Pr*6842), *Pseudoalteromonas denitrificans* (*Pd*6059), and *Pseudoalteromonas tunicata* (*Pt*D2). The relationship between *pigE* and *tamH* is discussed in the Further insight via functional genomics and DNA manipulation **(C)** Mapping of homologous cyclizing oxygenases in *Streptomyces coelicolor* [*Sc*A3(2)], *Pseudoalteromonas rubra* (*Pr*6842) and *Pseudoalteromonas* *tunicata* (*Pt*D2).

*Pseudoalteromonas* bacterial strains synthesize prodiginine analogs including prodigiosin, cycloprodigiosin, and 2-(*p*-hydroxybenzyl) prodigiosin (Figure 1A). These pigments possess a tripyrrole structure composed of two moieties: 2-methyl-3-*n*-amyl-pyrrole (MAP) and 4-methoxy-2,2′-bipyrrole-5-carbaldehyde (MBC). These analogs demonstrate antibacterial, anticancer, and immunosuppressive bioactivity and each analog exhibits its own specific bioactive properties. The specificity among these microbial pigments emphasizes their potential biomedical application.

#### Prodigiosin (*pig*) Biosynthetic Pathway

Prodigiosin biosynthesis was first characterized in the γ-proteobacterium, *Serratia* *marcescens* and later characterized in *Pseudoalteromonas rubra* (Fehér et al., 2008; Vynne et al., 2011). This model defines a unidirectional polycistronic *pig* gene cluster consisting of two enzymatic pathways (Harris et al., 2004; Figure 1A).

The unique pathway involves MAP biosynthesis from 2-octenal (**1)** and is composed of genes *pigB*, *pigD*, and *pigE*. First, PigD, in the presence of coenzyme thiamine pyrophosphate (TPP), catalyzes the addition of pyruvate (**2**) to 2-octenal (**1**). The formation of 3-acetyloctanal (**3**) occurs with the release of CO_2_, followed by the transfer of an amino group to the aldehyde by PigE, and cyclization to form H_2_MAP (**4**). PigB catalyzes further oxidation to form MAP (**5**) (Goldschmidt and Williams, 1968; Harris et al., 2004; Williamson et al., 2005; Garneau-Tsodikova et al., 2006).

The common pathway in most prodiginine-producing microorganisms is MBC biosynthesis. This pathway is composed of seven genes (*pigA*, *pigF*-*J*, *pigL*, and *pigM*). 4′-phosphopantetheinyl transferase (PigL) activates peptidyl carrier protein (PCP) domain of PigG from apo to holo form through the addition of the 4′-phosphopantetheinyl group. PigI and ATP catalyze the transfer of the L-prolyl group of L-proline (**6)** to the thiol group of phosphopantetheine to form a L-prolyl-*S*-PCP intermediate (**7**). PigA oxidizes the intermediate to pyrrolyl-2-carboxyl-*S*-PCP (**8**). This proceeds with a polyketide-type chain extension, which starts with the transfer of the pyrrole-2-carboxyl group from PigG to the cysteine active site of PigJ, generating a pyrrole-2-carboxyl thioester (**9**). Separately, the ACP domains of PigH are phosphopantetheinylated, providing binding sites for the malonyl group of malonyl-CoA (**10**). The bound malonyl (**11)** then undergoes decarboxylation, resulting in a subsequent condensation with the pyrrole-2-carboxyl thioester attached to PigJ and forms pyrrolyl-β-ketothioester on PigH (**12**). PigH catalyzes another decarboxylation between serine and pyrrolyl-β-ketothioester to generate 4-hydroxy-2,2′-bipyrrole-5-methanol (HBM) (**13**) (Harris et al., 2004; Garneau-Tsodikova et al., 2006). The alcohol group of HBM is oxidized by PigM to make 4-hydroxy-2,2′-bipyrrole-5-carbaldehyde (HBC) (**14**). The final step of MBC (**15**) biosynthesis involves the methylation of the HBC hydroxyl group by methyltransferase (PigF) and oxidoreductase (PigN) (Feitelson and Hopwood, 1983; Williamson et al., 2005).

Once MAP (**5**) and MBC (**15**) are synthesized, PigC uses ATP to facilitate the terminal condensation of these two pyrroles to form prodigiosin (**16**). PigC has shown sequence similarity to an ATP-binding and phosphoryl transfer domains at the N- and C- terminus, respectively (Morrison, 1966; Williams, 1973).

#### Prodigiosin Bioactivity

Prodigiosin demonstrated a variety of biological activities, making it a candidate for pharmaceutical development. It exhibited activity against chloroquine-resistant strains of *Plasmodium falciparum*, which causes cerebral malaria (Singh and Shekhawat, 2012). Although previously considered to have selective activity against Gram-positive bacteria, prodigiosin has shown broad-spectrum antimicrobial activity against bacterial species including *Bacillus subtilis*, *Escherichia coli, Enterobacter aerogenes*, *Enterococcus avium*, *Pseudomonas aeruginosa*, *Staphylococcus aureus*, *Staphylococcus saprophyticus*, *Streptococcus pyogenes* and *Candida albicans* (Lapenda et al., 2014; Darshan and Manonmani, 2015; Danevčič et al., 2016a,b; Suryawanshi et al., 2017). Prodigiosin has a variety of mechanisms for antibacterial activity, including inducing autolysin production in *B. cereus* and inhibiting biofilm production in *P. aeruginosa* by stimulating the production of reactive oxygen species (ROS) (Danevčič et al., 2016a; Kimyon et al., 2016). Furthermore, prodigiosin demonstrated algicidal activity against *Phaeocystis globola* via influx of ROS, mitigating harmful algal blooms (Zhang et al., 2017).

Prodigiosin also presented toxigenic activity against chick embryos (Kalesperis et al., 1975) and exhibited cytotoxic activity via oxidative DNA cleavage, particularly against melanoma and liver cancer cells (Manderville, 2001). Additionally, prodigiosin induced apoptosis in human hematopoietic cancer cell lines including acute T-cell leukemia, promyelocytic leukemia, myeloma, Burkitt lymphoma cells, and B- and T-cells from B-cell chronic lymphocytic leukemia (Campàs et al., 2003). It also exhibited cytotoxicity against human gastric and colon cancer cells, and the V79 fibroblast cell line though a specific mechanism of action has yet to be determined (Da Silva Melo et al., 2000; Campàs et al., 2003). However, it does not exhibit genotoxicity, making it a candidate in pharmaceutical development (Guryanov et al., 2013). Trypanocidal activity was also characterized against protozoan *Trypanosoma cruzi* which causes Chagas disease (Da Silva Melo et al., 2000).

Furthermore, prodigiosin demonstrated selective immunosuppressive properties in the murine models (Tsuji et al., 1990). It was capable of suppressing T-cell proliferation without affecting interleukin-2, transferrin, and antibody expression or response. These selective properties make prodigiosin a prospective probe for the cytotoxic T-lymphocyte activation pathway.

#### Cycloprodigiosin Biosynthetic Pathway

Recently, prodiginine classes were characterized in the marine bacterium genus *Pseudoalteromonas*, specifically, *Pseudoalteromonas denitrificans*, *Pseudoalteromonas rubra*, and *Pseudoalteromonas tunicata*. Cycloprodigiosin (cPrG) (**22**) is a red-pigmented secondary metabolite that was isolated from the marine bacteria *P. denitrificans* and *P. rubra* (Kim et al., 1999; Xie et al., 2012). The key structural difference between cPrG and prodigiosin is the cyclization between the C-3 pentyl group and the C-4 carbon on pyrrole ring C of MAP (**5**).

Cyclic prodiginines and their respective oxygenase for cyclization were identified. In *Streptomyces*, *redG* and *mcpG* encode oxygenases that cyclize undecylprodiginine to form butyl-meta-cycloheptylprodiginine and metacycloprodigiosin, respectively (Kimata et al., 2017). Recent studies characterized a homologous gene (PRUB680), encoding an alkylglycerol monooxygenase-like protein away from the *pig* biosynthetic gene cluster (de Rond et al., 2017). This enzyme shows regiospecificity via C-H activation prompting the cyclization of prodigiosin and formation of cPrG (**22**).

It is still unclear what sort of advantages, if any, come with prodigiosin cyclization (de Rond et al., 2017). Whether this favorability is dependent on structural stability, heightened bioactivity, or a combination of both has yet to be determined.

#### Cycloprodigiosin Bioactivity

cPrG showed higher antimalarial activity against *Plasmodium berghei* cells than chloroquine and its derivatives (Kim et al., 1999). Other cPrG analogs such as metacycloprodigiosin hydrochloride also demonstrated antimalarial activity against a multidrug-resistant strain of *P. falciparum* (Isaka et al., 2002). cPrG exhibited antimicrobial activity against *S. aureus*, *E. coli*, and *C. albicans* (Johnson et al., 2015; Offret et al., 2016).

cPrG was also characterized as an immunosuppressant through its ability to induce apoptosis of activated murine splenic T-cells, acute human T-cell leukemia, promyelocytic leukemia, human and rat hepatocellular cancer, human breast cancer, and TNF-stimulated human cervix carcinoma (Darshan and Manonmani, 2015).

#### 2-(*p*-Hydroxybenzyl) Prodigiosin Biosynthetic Pathway

2-(*p*-hydroxybenzyl) prodigiosin (HBPG) (**23**) was isolated from the marine bacterium *P. rubra* (Fehér et al., 2008; Offret et al., 2016). The structural difference between HBPG (**23**) and prodigiosin (**16**) is the *para*-addition of phenol to the C-10 carbon of pyrrole ring A of MBC (**15**), suggesting variations to the MBC pathway and homologs in the MAP pathway and terminal condensation step. Currently, there is no literature describing HBPG biosynthesis. Difficulties in characterization arise when biosynthetic genes are not clustered together, like that of the cPrG pathway. As shown by de Rond et al. (2017) elucidation of the complete biosynthetic pathway may rely on mining for homologous genes in other bacterial genera that function, similarly.

#### 2-(*p*-Hydroxybenzyl) Prodigiosin Bioactivity

Due to HBPG’s exclusivity in only a few *P. rubra* strains, little information has been collected regarding its bioactivity. Like other prodiginines, HBPG demonstrated antimicrobial activity against *S. aureus*, methicillin-resistant *S. aureus* (MRSA), *E. coli*, and *C. albicans* (Fehér et al., 2008; Hu et al., 2016; Offret et al., 2016).

Cytotoxic activity of HBPG was also characterized against SKOV-3, a human ovarian adenocarcinoma cell line, via topoisomerase I inhibition. These results were indistinguishable from the prodigiosin control (Fehér et al., 2008). Additional bioassays will need to be conducted to uncover the significance of the *p*-hydroxylbenzyl group.

#### Tambjamine (*tam*) Biosynthetic Pathway

*Pseudoalteromonas tunicata* has yielded tambjamine, a yellow bipyrrolic bacterial pigment. Despite its divergence from the tripyrrolic structure, tambjamines were characterized to be structurally-related to prodiginines and share sequence homology to the *pig* biosynthetic pathway (Burke et al., 2007). Like the prodiginine family, tambjamine shares the MBC-moiety and contains the homologous genes responsible for its biosynthesis, however, it lacks the MAP-moiety, instead having an enamine group. Two types of tambjamines were characterized within *P. tunicata* – tambjamine and tambjamine YP1. The major difference between the two analogs is the substituent group attached to C-4: a methoxy group in tambjamine, where tambjamine YP1 has a methyl group (Franks et al., 2006).

Novel to the *tam* pathway is the enamine biosynthetic pathway. This pathway is composed of three characterized genes (*tamT, tamH, afaA*) and begins with dodecenoic acid (**17**). Until recently, *afaA* was thought to initiate the enamine biosynthesis (Burke et al., 2007). However, it was later determined that acyl CoA synthetase (TamA) is actually responsible for activating dodecenoic acid (**17**) (Marchetti et al., 2018). The activated fatty acid (**18**) undergoes oxidation by dehydrogenase (TamT), introducing a π-bond to C-3 carbon of the fatty acyl side chain (**19**). Reductase/aminotransferase (TamH) facilitates the reduction of the CoA-ester (**19**) and transamination to dodec-3-en-1-amine (**20**). TamQ facilitates the condensation of MBC (**15**) and enamine (**20**) to form tambjamine (**21**) (Burke et al., 2007).

#### Tambjamine Bioactivity

Tambjamines exhibit antifungal, antifouling, and antimicrobial activity against *S. aureus, E. coli, C. albicans*, and *Malassezia furfur* (Holmström et al., 2002; Franks et al., 2006; Pinkerton et al., 2010). In addition, the production of tambjamine had inhibitory bioactivity against the bacterivorous nematode *Caenorhabditis elegans* (Ballestriero et al., 2010).

Since the characterization of the *tam* pathway, tambjamines analogs were chemically synthesized to explore the antimicrobial and cytotoxic properties associated with the substitution of various alkyl and bromo groups (Pinkerton et al., 2010). Synthetic tambjamine analogs that contain aromatic enamine groups demonstrate efficient transmembrane anion transportation of chloride and bicarbonate (Hernando et al., 2014). These cytotoxic transport properties are a promising treatment of cancer cells. Unlike normal tissue cells, cancer cells contain a reversed pH gradient with alkaline intracellular and acidic extracellular conditions. These conditions provide tumor cells protection from acid-induced apoptosis, allowing them to proliferate (Castaing et al., 2001; Sharma, 2015). Introducing highly effective anion-selective ionophores, like tambjamine, can regulate cellular ion homeostasis by affecting the intracellular pH, triggering mitochondrial dysfunction and lysosomal deacidification, leading to necrosis in lung cancer cell lines and cancer stem cells (Rodilla et al., 2001).

## Evolutionary and Ecological Significance

Recent literature suggests that the *pig* pathway can undergo horizontal gene transfer, which may explain the homology between prodigiosin gene clusters in *Serratia* and *Pseudoalteromonas* (Coulhurst et al., 2006; Williamson et al., 2006; Figure 1B). Although *Serratia* is primarily terrestrial, the genus’s presence in marine and aquatic environments has been reported and may be substantial enough to interact with the exclusively marine *Pseudoalteromonas* (Joyner et al., 2014). The versatile bioactivity of prodiginines implies an integral role in outcompeting other microorganisms for valuable resources.

Furthermore, a majority of the *Pseudoalteromonas* species are associated with eukaryotic hosts (Holmström and Kjelleberg, 1999). From the three species described, *P. denitrificans* and *P. rubra* were isolated from seawater samples, while *P. tunicata* was characterized in marine algae and tunicates (Bowman, 2007). There is a close association between pigmented *Pseudoalteromonas* species and marine macroorganisms (Zheng et al., 2006; Sakai-Kawada et al., 2016). One study isolated *P. tunicata* from a healthy adult tunicate and *Ulva lactuca*, a common green alga; both hosts did not independently produce antifouling compounds. *P. tunicata* produced these bioactive compounds, preventing colonization of algal spores, marine invertebrate larvae, protozoa, bacteria, and fungi, supporting the hypothesis that *Pseudoalteromonas* species may act as a chemical defense mechanism for its associated host (Holmström et al., 2002; Franks et al., 2006). Although the *Pseudoalteromonas* genus is associated with a wide array of bioactivities that may provide protection for their respective hosts, difficulties still arise when determining the precise ecological role, they play.

## Further Insight Via Functional Genomics and DNA Manipulation

With current breakthroughs in functional genomics, one can now identify homologous prodiginine biosynthetic gene clusters within *Pseudoalteromonas* to characterize the pathway. 9 of the 200+ *Pseudoalteromonas* genomes submitted on NCBI are known to synthesize prodiginines. Of the 9 genomes, 8 belong to *P. rubra* and only 1 to *P. denitrificans*. Genome sequencing and prodiginine biosynthesis-directed annotation can provide more insight into homologous pathways (Figure 1B and Table 1). Preliminary genome analysis in both *Pseudoalteromonas* species revealed a gene cluster homologous to the prodiginine biosynthetic gene cluster in *Serratia. P. rubra*’*s* gene cluster averaged 63% homology to *Serratia*, while *P. denitrificans* averaged 54% (Xie et al., 2012; Li et al., 2016). Although *P. tunicata*’*s* tambjamine biosynthetic gene cluster shares a similar MBC pathway with prodiginine biosynthesis in other *Pseudoalteromonas* species, it showed low identity averaging 46% homology (Burke et al., 2007). However, sequence homology extended beyond the MBC pathway. Putative aminotransferase (*tamH*) responsible for enamine biosynthesis in *P. tunicata* showed 40% identity to an aminotransferase (*pigE*) responsible for MAP biosynthesis in *Serratia* and the other two *Pseudoalteromonas* species (Burke et al., 2007).

**Table 1:** Predicted function of genes involved in prodiginine and tambjamine biosynthesis and their homology among *Serratia marcescens* (*Sm*39006), *Pseudoalteromonas* *rubra* (*Pr*6842), *Pseudoalteromonas denitrificans* (*Pd*6059), and *Pseudoalteromonas tunicata* (*Pt*D2).

Biosynthetic pathway	Predicted protein function	*pig* gene cluster (*Sm*39006)	*tam* gene cluster (*Pt*D2)	ORFs (*Pd*6059/ *Pr*6842)
MAP	Oxidoreductase	*pigB*	*–*	ORF2
MAP	Thiamine diphosphate dependent-3-acetyloctanal synthase	*pigD*	*–*	ORF4
MAP/Enamine	Aminotransferase	*pigE*	*tamH*^a^	ORF5
MBC	L-prolyl-PCP dehydrogenase	*pigA*	*tamG*	ORF1
MBC	S-adenosyl-L-methionine-dependent methyltransferase	*pigF*	*tamP*	ORF6
MBC	Peptidyl carrier protein	*pigG*	*tamB*	ORF7
MBC	HBM synthase/aminotransferase	*pigH*	*tamD*	ORF8
MBC	L-prolyl-AMP ligase	*pigI*	*tamE*	ORF9
MBC	Pyrrolyl-β-ketoacyl-ACP synthase	*pigJ*	*tamF*	ORF10
MBC	Hypothetical protein	*pigK*	*tamR*	ORF11
MBC	Phosphopantetheinyl transferase	*pigL*	*tamS*	ORF12
MBC	HBM oxidase/dehydrogenase	*pigM*	*tamJ*	ORF13
MBC	Oxidoreductase	*pigN*	*–*	*–*
Terminal Condensation	Terminal Condensing Enzyme	*pigC*	*tamQ*	ORF3
Cyclization	Putative oxidase	*–*	*tamC*	PRUB680 ^b^
Enamine	AMP binding protein	*–*	*tamA*	*–*
Enamine	Putative dehydrogenase	*–*	*tamT*	*–*
Tambjamine	Hypothetical protein	*–*	*tamK*	*–*
Tambjamine	Putative permease	*–*	*tamL*	*–*
Tambjamine	Putative permease	*–*	*tamM*	*–*
Tambjamine	Putative ABC transporter	*–*	*tamN*	*–*
Tambjamine	Hypothetical protein	*–*	*tamO*	*–*


*^a^ Although there is sequence similarity to pigE in MAP biosynthesis, tamH is associated with enamine biosynthesis. ^b^ PRUB680 is characterized only in P. rubra.*

As previously discussed, there are limitations to relying solely on functional genomics. Genes outside of the primary gene cluster, as demonstrated with PRUB680 in *P. rubra*, play a key role in both regulating and biosynthesizing prodiginine analogs. However, PRUB680 showed low sequence homology to *redG* in *Streptomyces* at 46% (Figure 1C). Although genome sequencing and rapid annotation via localized alignment tools are useful for characterizing numerous biosynthetic gene clusters, proper genetic manipulation of each individual ORF is necessary to confirm function. As previously discussed, Marchetti et al. (2018). demonstrated that *tamA* was responsible for the initiation of enamine biosynthesis, further feeding into tambjamine biosynthesis. That is not to say that *afaA* does not still play a role in tambjamine biosynthesis.

Several molecular tools have been used to manipulate *Pseudoalteromonas*’ genome and explore their numerous biological pathways. Egan et al. (2002) and Huang et al. (2012) both proved that the *Pseudoalteromonas* genome can be manipulated via Tn10 random mutagenesis, however, this method requires screening hundreds to thousands of mutants before isolating one that disrupts the gene of interest. de Rond et al. (2017) and Wang et al. (2015) utilized a site-directed approach in manipulating *P. rubra*’*s* genome via suicide and expression shuttle vectors, respectively. Recently, *Pseudoalteromonas* phages and prophages belonging to the myoviridae, podoviridae, and siphoviridae families have been isolated and sequenced several instances Duhaime et al., 2017. characterized a Mu-type sipho-myo hybrid capable of incorporating itself into the host genome as well as several phages containing proteins that can control host stress response. Given the proper delivery system, these bacteriophages could prove to be powerful tools for DNA manipulation within *Pseudoalteromonas*.

In conclusion, a more holistic approach to understanding bioactivity and gene regulation will lay the foundation for answering overarching questions concerning pigmented *Pseudoalteromonas* species such as their ecological and overall roles within the marine microbiome.

## Author Contributions

FS-K and JA contributed to the conception of the focus for the study. FS-K contributed to the compilation of all sections, figure and table design, and wrote the first draft of the manuscript. CI performed the compilation of biological activity, ecological and evolutionary significance sections. KH contributed to the biosynthetic pathways and biological activity sections. All authors contributed to revision, read and approved the submitted version of the manuscript.

## Conflict of Interest Statement

The authors declare that the research was conducted in the absence of any commercial or financial relationships that could be construed as a potential conflict of interest.
